# The genome sequence of the grey gurnard, *Eutrigla gurnardus *(Linnaeus, 1758)

**DOI:** 10.12688/wellcomeopenres.22453.1

**Published:** 2024-06-11

**Authors:** Rachel Brittain, Patrick Adkins, Joanna Harley, Marine Biological Association Genome Acquisition Lab, Darwin Tree of Life Barcoding collective, Wellcome Sanger Institute Tree of Life Management, Samples and Laboratory team, Wellcome Sanger Institute Scientific Operations: Sequencing Operations, Wellcome Sanger Institute Tree of Life Core Informatics team, Tree of Life Core Informatics collective, Darwin Tree of Life Consortium

**Affiliations:** 1 The Marine Biological Association, Plymouth, England, UK

**Keywords:** Eutrigla gurnardus, grey gurnard, genome sequence, chromosomal, Scorpaeniformes

## Abstract

We present a genome assembly from an individual *Eutrigla gurnardus* (the grey gurnard; Chordata; Actinopteri; Scorpaeniformes; Triglidae). The genome sequence is 680.5 megabases in span. Most of the assembly is scaffolded into 24 chromosomal pseudomolecules. The mitochondrial genome has also been assembled and is 16.51 kilobases in length.

## Species taxonomy

Eukaryota; Opisthokonta; Metazoa; Eumetazoa; Bilateria; Deuterostomia; Chordata; Craniata; Vertebrata; Gnathostomata; Teleostomi; Euteleostomi; Actinopterygii; Actinopteri; Neopterygii; Teleostei; Osteoglossocephalai; Clupeocephala; Euteleosteomorpha; Neoteleostei; Eurypterygia; Ctenosquamata; Acanthomorphata; Euacanthomorphacea; Percomorphaceae; Eupercaria; Perciformes; Triglioidei; Triglidae; *Eutrigla*; *Eutrigla gurnardus* (Linnaeus, 1758) (NCBI:txid426098).

## Background

**Figure 1.**
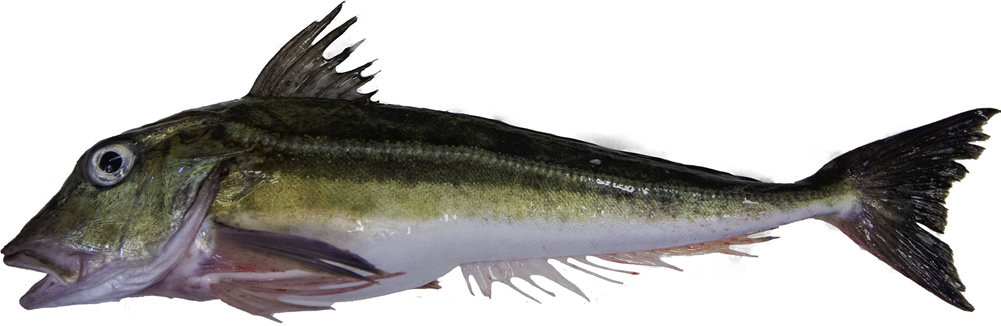
Photograph of *Eutrigla gurnardus* (not the specimen used for genome sequencing) (image by Clumpus).

*Eutrigla gurnardus,* commonly called the Grey Gurnard, is a demersal teleost fish belonging to the family Triglidae ( Figure 1). It is distributed in shelf seas and coastal waters from Norway, Greenland and Iceland in the north, to as far south as Mauritania and the Azores, including the Mediterranean and black sea ( Blanc & Hureau, 1979; Froese & Pauly, 2023; Hureau, 1986; Neudecker & Stein, 2011). *E. gurnardus* is commonly found on sandy/muddy-sand bottoms around the entire of the UK. It is traditionally more common in southern sites in the UK. However, its distribution has been shown to be changing in response to climatic changes ( Perry *et al.,* 2005).

*E. gurnardus* is typically grey or brownish in colour (occasionally dull red) and whitish ventrally. As with other members of the Triglidae family it has modified pectoral fins, the first three rays of which form slender tactile processes. It can be distinguished from other members of UK Triglidae by having short pectoral fins which do not reach as far back as the anal fin and by having spines along its lateral line.

A significant predator around much of the UK ( Daan *et al*., 1990), *E. gurnardus* feeds on a range of invertebrate species (mostly crustaceans) and small fish ( Weinert *et al.*, 2010). During competition for food, it has been shown to make vocalisations in the form of grunts, growls and knocks ( Amorim *et al.*, 2004).

In the past gurnards have not been split into their respective species when landed and have instead been treated as a single group. It has therefore been difficult to interpret fisheries data when trying to regard individual gurnard species. Even today, due to the low economic value of this species, it is likely that landing data does not accurately reflect the actual numbers of individuals caught, with the majority of gurnards being discarded after being caught alongside target demersal species such as flatfish ( Enever *et al.,* 2007; Enever *et al.,* 2009; ICES, 2006; ICES, 2012).

This genome represents the first of its kind for this *Eutrigla gurnardus*. It was sequenced as part of the Darwin Tree of Life Project, a collaborative effort to sequence all named eukaryotic species in the Atlantic Archipelago of Britain and Ireland.

## Genome sequence report

The genome was sequenced from one *Eutrigla gurnardus* collected using a trawl in Whitsand Bay, English Channel, UK (latitude 50.26, longitude –3.95). A total of 34-fold coverage in Pacific Biosciences single-molecule HiFi long reads was generated. Primary assembly contigs were scaffolded with chromosome conformation Hi-C data. Manual assembly curation corrected 24 missing joins or mis-joins and removed 3 haplotypic duplications, reducing the scaffold number by 4.20%.

**Figure 2.**
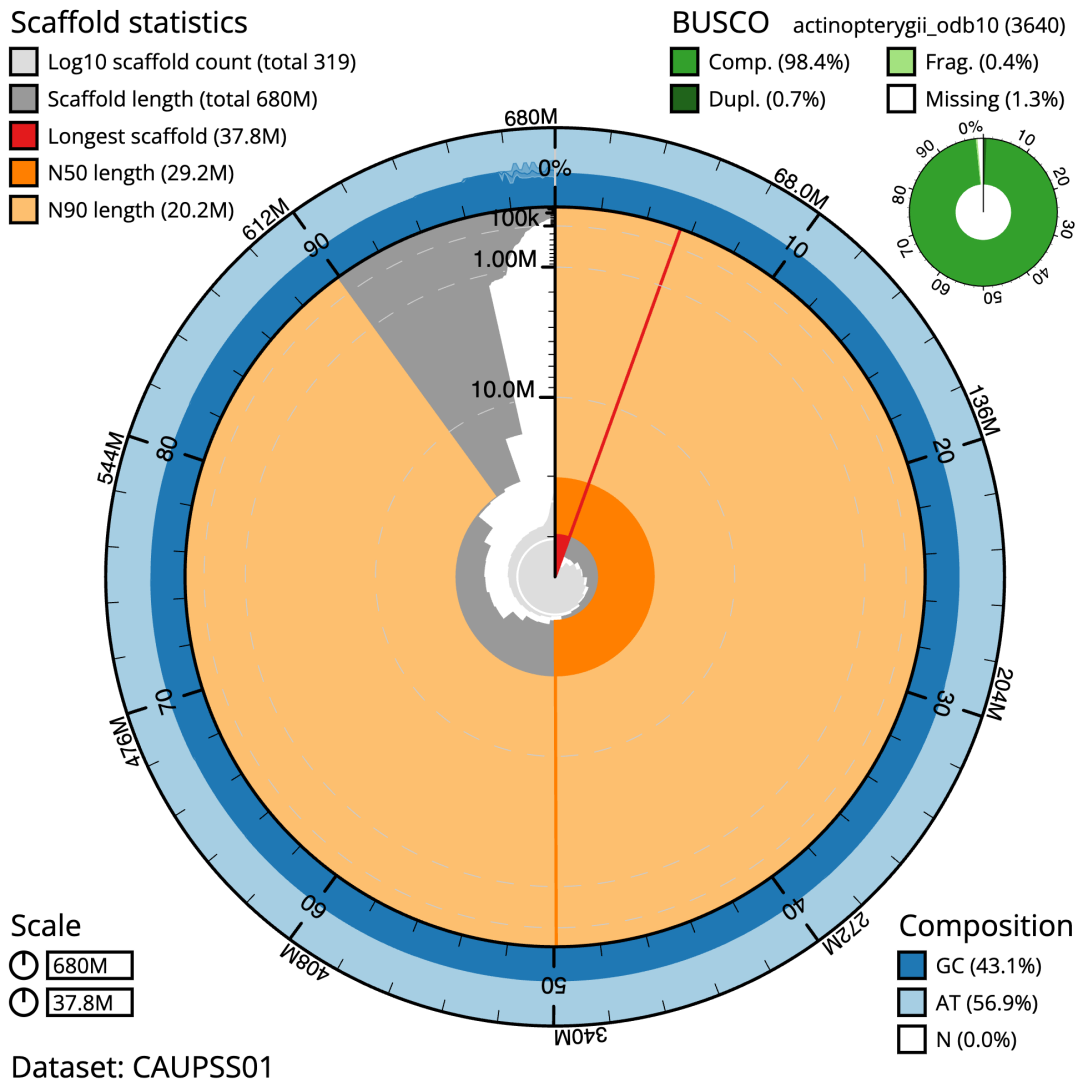
Genome assembly of *Eutrigla gurnardus*, fEutGur1.1: metrics. The BlobToolKit snail plot shows N50 metrics and BUSCO gene completeness. The main plot is divided into 1,000 size-ordered bins around the circumference with each bin representing 0.1% of the 680,483,561 bp assembly. The distribution of scaffold lengths is shown in dark grey with the plot radius scaled to the longest scaffold present in the assembly (37,750,515 bp, shown in red). Orange and pale-orange arcs show the N50 and N90 scaffold lengths (29,198,404 and 20,162,171 bp), respectively. The pale grey spiral shows the cumulative scaffold count on a log scale with white scale lines showing successive orders of magnitude. The blue and pale-blue area around the outside of the plot shows the distribution of GC, AT and N percentages in the same bins as the inner plot. A summary of complete, fragmented, duplicated and missing BUSCO genes in the actinopterygii_odb10 set is shown in the top right. An interactive version of this figure is available at https://blobtoolkit.genomehubs.org/view/CAUPSS01/dataset/CAUPSS01/snail.

**Figure 3.**
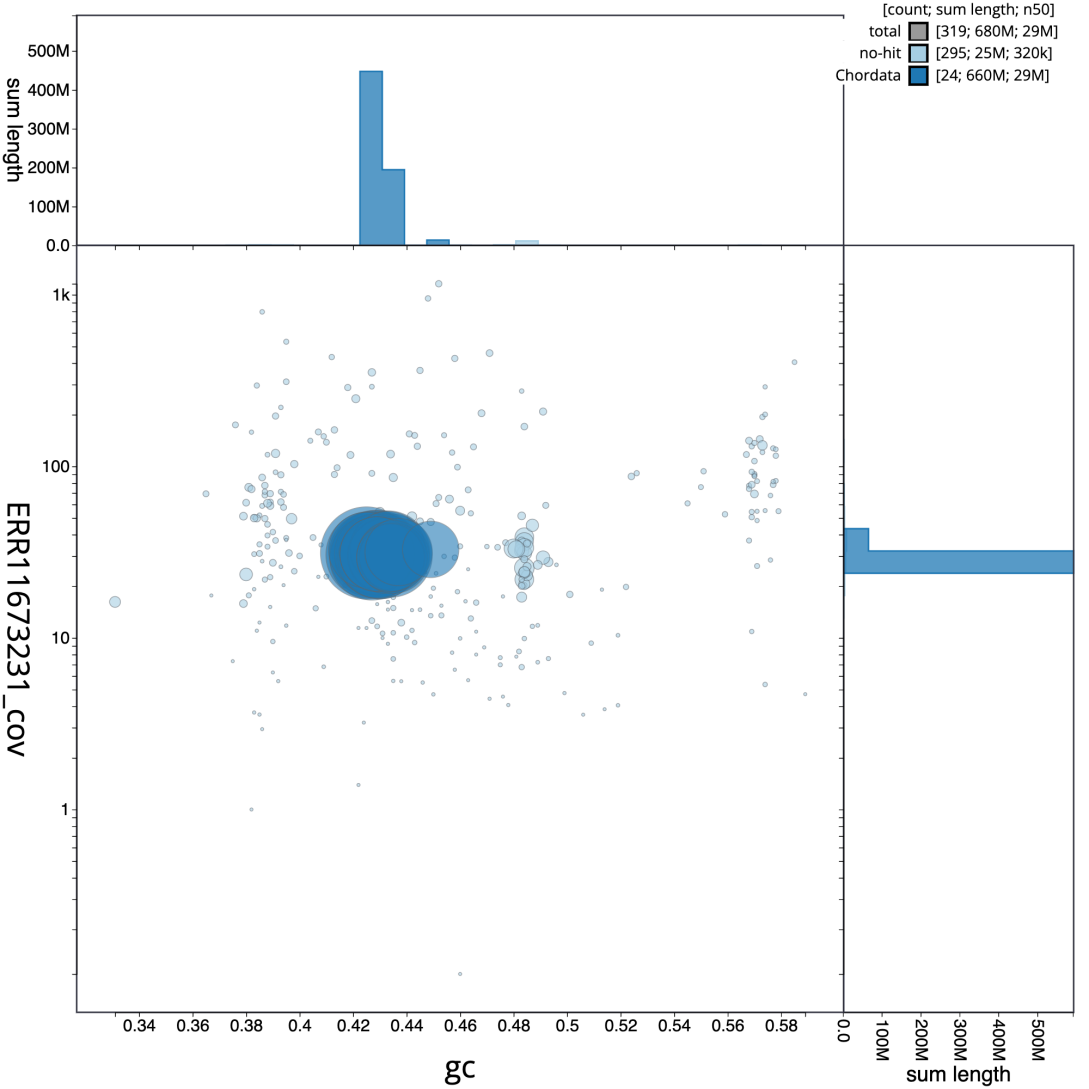
Genome assembly of *Eutrigla gurnardus*, fEutGur1.1: BlobToolKit GC-coverage plot. Sequences are coloured by phylum. Circles are sized in proportion to sequence length. Histograms show the distribution of sequence length sum along each axis. An interactive version of this figure is available at https://blobtoolkit.genomehubs.org/view/CAUPSS01/dataset/CAUPSS01/blob.

**Figure 4.**
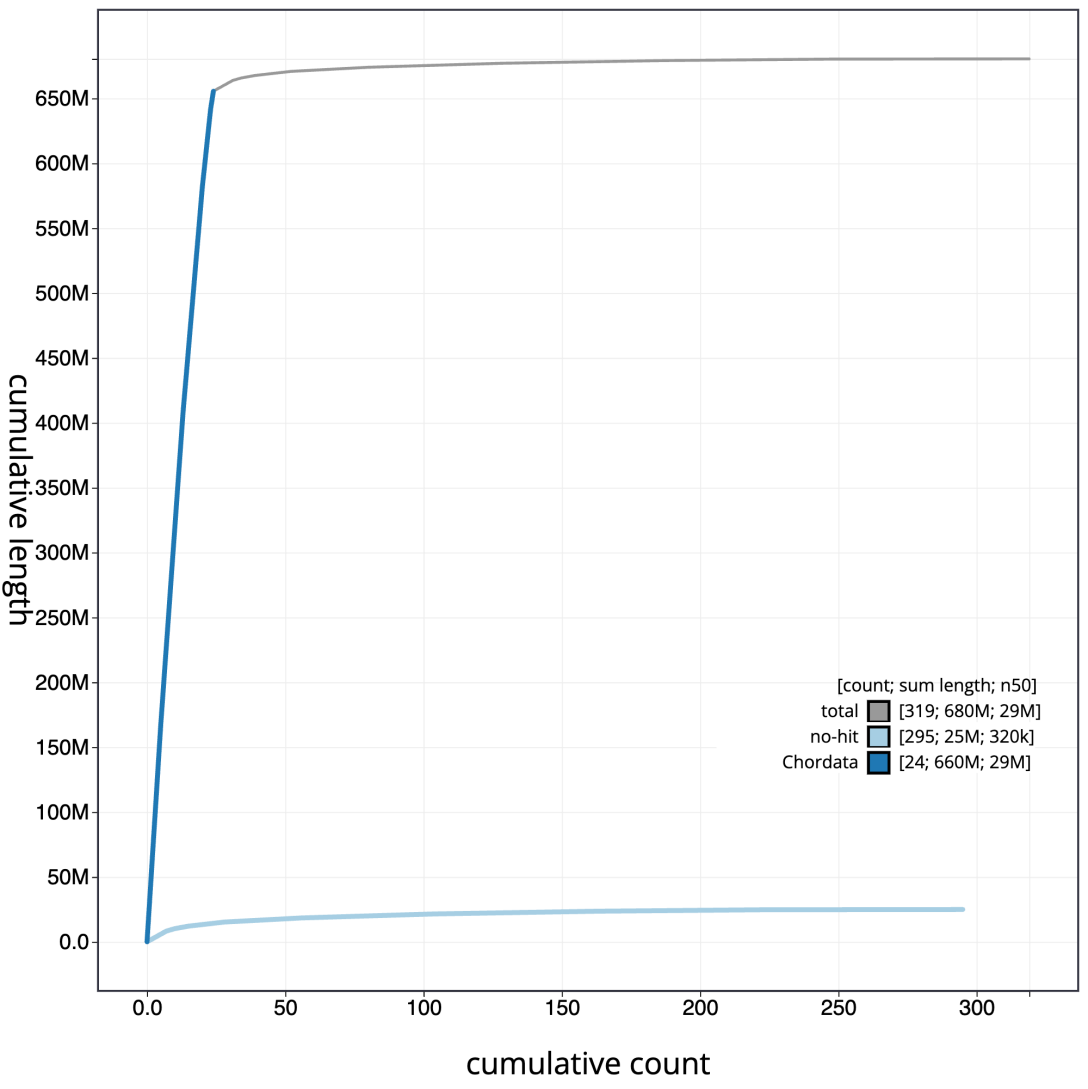
Genome assembly of *Eutrigla gurnardus*, fEutGur1.1: BlobToolKit cumulative sequence plot. The grey line shows cumulative length for all sequences. Coloured lines show cumulative lengths of sequences assigned to each phylum using the buscogenes taxrule. An interactive version of this figure is available at https://blobtoolkit.genomehubs.org/view/CAUPSS01/dataset/CAUPSS01/cumulative.

**Figure 5.**
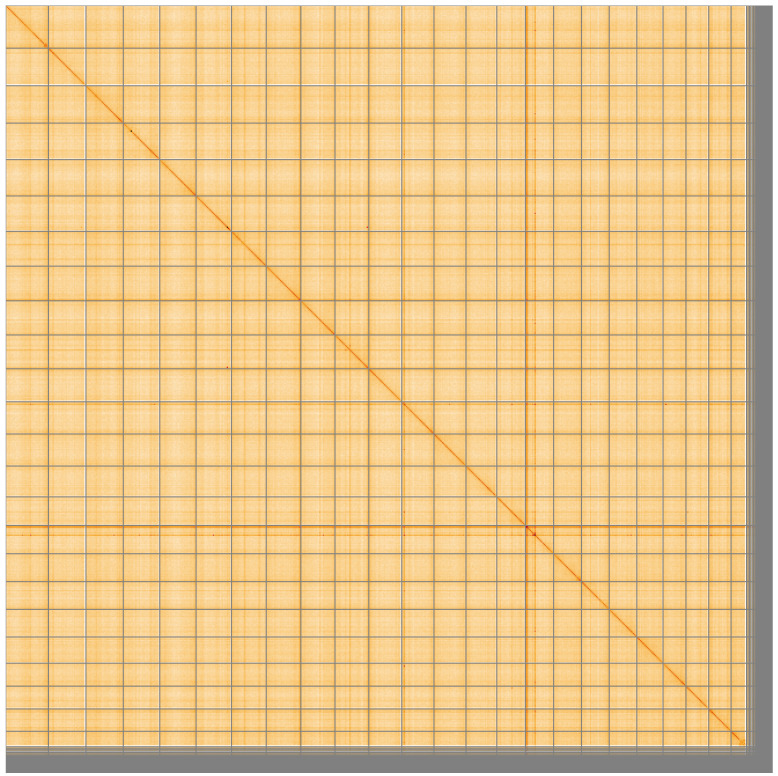
Genome assembly of *Eutrigla gurnardus*, fEutGur1.1: Hi-C contact map of the fEutGur1.1 assembly, visualised using HiGlass. Chromosomes are shown in order of size from left to right and top to bottom. An interactive version of this figure may be viewed at https://genome-note-higlass.tol.sanger.ac.uk/l/?d=T0CMcjkMSfGbGvPjIDJGGg.

The final assembly has a total length of 680.5 Mb in 318 sequence scaffolds with a scaffold N50 of 29.2 Mb ( Table 1). The snail plot in Figure 2 provides a summary of the assembly statistics, while the distribution of assembly scaffolds on GC proportion and coverage is shown in Figure 3. The cumulative assembly plot in Figure 4 shows curves for subsets of scaffolds assigned to different phyla. Most (96.33%) of the assembly sequence was assigned to 24 chromosomal-level scaffolds. Chromosome-scale scaffolds confirmed by the Hi-C data are named in order of size ( Figure 5; Table 2). While not fully phased, the assembly deposited is of one haplotype. Contigs corresponding to the second haplotype have also been deposited. The mitochondrial genome was also assembled and can be found as a contig within the multifasta file of the genome submission.

**Table 1.** Genome data for *Eutrigla gurnardus*, fEutGur1.1.

Project accession data		
Assembly identifier	fEutGur1.1	
Species	*Eutrigla gurnardus*	
Specimen	fEutGur1	
NCBI taxonomy ID	426098	
BioProject	PRJEB64070	
BioSample ID	SAMEA111562159	
Isolate information	fEutGur1, gill tissue (PacBio DNA, Illumina Hi-C and RNA sequencing)	
Assembly metrics *		*Benchmark*
Consensus quality (QV)	55.6	*≥ 50*
*k*-mer completeness	99.99%	*≥ 95%*
BUSCO **	C:98.4%[S:97.7%,D:0.7%],F:0.4%, M:1.3%,n:3,640	*C ≥ 95%*
Percentage of assembly mapped to chromosomes	96.33%	*≥ 95%*
Sex chromosomes	None	*localised homologous pairs*
Organelles	Mitochondrial genome: 16.51 kb	*complete single alleles*
Raw data accessions		
PacificBiosciences Sequel IIe	ERR11673231	
Hi-C Illumina	ERR11679384, ERR11679385	
PolyA RNA-Seq Illumina	ERR11837500	
Genome assembly		
Assembly accession	GCA_963514095.1	
*Accession of alternate haplotype*	GCA_963514115.1	
Span (Mb)	680.5	
Number of contigs	872	
Contig N50 length (Mb)	2.4	
Number of scaffolds	318	
Scaffold N50 length (Mb)	29.2	
Longest scaffold (Mb)	37.75	

* Assembly metric benchmarks are adapted from column VGP-2020 of “Table 1: Proposed standards and metrics for defining genome assembly quality” from Rhie *et al.* (2021). ** BUSCO scores based on the actinopterygii_odb10 BUSCO set using version 5.3.2. C = complete [S = single copy, D = duplicated], F = fragmented, M = missing, n = number of orthologues in comparison. A full set of BUSCO scores is available at https://blobtoolkit.genomehubs.org/view/CAUPSS01/dataset/CAUPSS01/busco.

**Table 2.** Chromosomal pseudomolecules in the genome assembly of *Eutrigla gurnardus*, fEutGur1.

INSDC accession	Chromosome	Length (Mb)	GC%
OY741264.1	1	37.75	42.5
OY741265.1	2	33.21	42.5
OY741266.1	3	33.05	43.0
OY741267.1	4	32.29	43.0
OY741268.1	5	32.14	42.5
OY741269.1	6	31.33	42.5
OY741270.1	7	30.74	43.5
OY741271.1	8	30.68	43.5
OY741272.1	9	30.19	43.0
OY741273.1	10	29.9	43.0
OY741274.1	11	29.2	43.0
OY741275.1	12	28.47	43.0
OY741276.1	13	28.36	43.0
OY741277.1	14	27.27	43.0
OY741278.1	15	25.25	42.5
OY741279.1	16	25.05	43.5
OY741280.1	17	24.68	43.0
OY741281.1	18	24.52	43.0
OY741282.1	19	24.42	43.0
OY741283.1	20	23.17	43.5
OY741284.1	21	20.33	43.5
OY741285.1	22	20.16	43.5
OY741286.1	23	19.75	43.5
OY741287.1	24	13.66	45.0
OY741288.1	MT	0.02	48.5

The estimated Quality Value (QV) of the final assembly is 55.6 with *k*-mer completeness of 99.99%, and the assembly has a BUSCO v5.3.2 completeness of 98.4% (single = 97.7%, duplicated = 0.7%), using the actinopterygii_odb10 reference set ( *n* = 3,640).

Metadata for specimens, BOLD barcode results, spectra estimates, sequencing runs, contaminants and pre-curation assembly statistics are given at https://tolqc.cog.sanger.ac.uk/darwin/fish/Eutrigla_gurnardus/.

## Methods

### Sample acquisition and nucleic acid extraction

A *Eutrigla gurnardus* specimen (specimen ID MBA-211118-001A, ToLID fEutGur1) was collected from Whitsand Bay, English Channel, UK (latitude 50.26, longitude –3.95) on 29 April 2022. The specimen was taken from its habitat of sand and broken shell using an otter trawl deployed from RV Sepia. The specimen was identified by Rachel Brittain, Patrick Adkins and Joanna Harley (Marine Biological Association) based on gross morphology. The fish was first anesthetised and then overdosed using Aquased (2-phenoxyethanol). Destruction of the brain was used as a secondary method to ensure the animal was deceased before tissue sampling took place as in accordance with Schedule 1 methodology under the home office licence. Samples taken from the animal were preserved on dry ice.

The workflow for high molecular weight (HMW) DNA extraction at the Wellcome Sanger Institute (WSI) Tree of Life Core Laboratory includes a sequence of core procedures: sample preparation; sample homogenisation, DNA extraction, fragmentation, and clean-up. The sample was prepared for extraction at the Tree of Life Core Laboratory: tissue from the gills of fEutGur1 sample was weighed and dissected on dry ice ( Jay *et al.*, 2023) and homogenised using a PowerMasher II tissue disruptor ( Denton *et al.*, 2023a).

HMW DNA was extracted in the WSI Scientific Operations core using the Automated MagAttract v2 protocol ( Oatley *et al.*, 2023). The DNA was sheared into an average fragment size of 12–20 kb in a Megaruptor 3 system with speed setting 31 ( Bates *et al.*, 2023). Sheared DNA was purified by solid-phase reversible immobilisation ( Strickland *et al.*, 2023): in brief, the method employs a 1.8X ratio of AMPure PB beads to sample to eliminate shorter fragments and concentrate the DNA. The concentration of the sheared and purified DNA was assessed using a Nanodrop spectrophotometer and Qubit Fluorometer and Qubit dsDNA High Sensitivity Assay kit. Fragment size distribution was evaluated by running the sample on the FemtoPulse system.

RNA was extracted from gill tissue of fEutGur1 in the Tree of Life Laboratory at the WSI using the RNA Extraction: Automated MagMax™ *mir*Vana protocol ( do Amaral *et al.*, 2023). The RNA concentration was assessed using a Nanodrop spectrophotometer and a Qubit Fluorometer using the Qubit RNA Broad-Range Assay kit. Analysis of the integrity of the RNA was done using the Agilent RNA 6000 Pico Kit and Eukaryotic Total RNA assay.

Protocols developed by the WSI Tree of Life laboratory are publicly available on protocols.io ( Denton *et al.*, 2023b).

### Sequencing

Pacific Biosciences HiFi circular consensus DNA sequencing libraries were constructed according to the manufacturers’ instructions. Poly(A) RNA-Seq libraries were constructed using the NEB Ultra II RNA Library Prep kit. DNA and RNA sequencing was performed by the Scientific Operations core at the WSI on Pacific Biosciences Sequel IIe (HiFi) and Illumina NovaSeq 6000 (RNA-Seq) instruments. Hi-C data were also generated from gill tissue of fEutGur1 using the Arima v2 kit. The Hi-C sequencing was performed using paired-end sequencing with a read length of 150 bp on the Illumina NovaSeq 6000 instrument.

### Genome assembly, curation and evaluation

Assembly was carried out with Hifiasm ( Cheng *et al.*, 2021) and haplotypic duplication was identified and removed with purge_dups ( Guan *et al.*, 2020). The assembly was then scaffolded with Hi-C data ( Rao *et al.*, 2014) using YaHS ( Zhou *et al.*, 2023). The assembly was checked for contamination and corrected using the TreeVal pipeline ( Pointon *et al.*, 2023). Manual curation was performed using JBrowse2 ( Diesh *et al.*, 2023), HiGlass ( Kerpedjiev *et al.*, 2018) and PretextView ( Harry, 2022). The mitochondrial genome was assembled using MitoHiFi ( Uliano-Silva *et al.*, 2023), which runs MitoFinder ( Allio *et al.*, 2020) or MITOS ( Bernt *et al.*, 2013) and uses these annotations to select the final mitochondrial contig and to ensure the general quality of the sequence.

### Final assembly evaluation

The final assembly was post-processed and evaluated with the three Nextflow ( Di Tommaso *et al.*, 2017) DSL2 pipelines “sanger-tol/readmapping” ( Surana *et al.*, 2023a), “sanger-tol/genomenote” ( Surana *et al.*, 2023b), and “sanger-tol/blobtoolkit” ( Muffato *et al.*, 2024). The pipeline sanger-tol/readmapping aligns the Hi-C reads with bwa-mem2 ( Vasimuddin *et al.*, 2019) and combines the alignment files with SAMtools ( Danecek *et al.*, 2021). The sanger-tol/genomenote pipeline transforms the Hi-C alignments into a contact map with BEDTools ( Quinlan & Hall, 2010) and the Cooler tool suite ( Abdennur & Mirny, 2020), which is then visualised with HiGlass ( Kerpedjiev *et al.*, 2018). It also provides statistics about the assembly with the NCBI datasets ( Sayers *et al.*, 2024) report, computes *k*-mer completeness and QV consensus quality values with FastK and MerquryFK, and a completeness assessment with BUSCO ( Manni *et al.*, 2021).

The sanger-tol/blobtoolkit pipeline is a Nextflow port of the previous Snakemake Blobtoolkit pipeline ( Challis *et al.*, 2020). It aligns the PacBio reads with SAMtools and minimap2 ( Li, 2018) and generates coverage tracks for regions of fixed size. In parallel, it queries the GoaT database ( Challis *et al.*, 2023) to identify all matching BUSCO lineages to run BUSCO ( Manni *et al.*, 2021). For the three domain-level BUSCO lineage, the pipeline aligns the BUSCO genes to the Uniprot Reference Proteomes database ( Bateman *et al.*, 2023) with DIAMOND ( Buchfink *et al.*, 2021) blastp. The genome is also split into chunks according to the density of the BUSCO genes from the closest taxonomically lineage, and each chunk is aligned to the Uniprot Reference Proteomes database with DIAMOND blastx. Genome sequences that have no hit are then chunked with seqtk and aligned to the NT database with blastn ( Altschul *et al.*, 1990). All those outputs are combined with the blobtools suite into a blobdir for visualisation.

All three pipelines were developed using the nf-core tooling ( Ewels *et al.*, 2020), use MultiQC ( Ewels *et al.*, 2016), and make extensive use of the Conda package manager, the Bioconda initiative ( Grüning *et al.*, 2018), the Biocontainers infrastructure ( da Veiga Leprevost *et al.*, 2017), and the Docker ( Merkel, 2014) and Singularity ( Kurtzer *et al.*, 2017) containerisation solutions.

Table 3 contains a list of relevant software tool versions and sources.

**Table 3.** Software tools: versions and sources.

Software tool	Version	Source
BEDTools	2.30.0	https://github.com/arq5x/bedtools2
Blast	2.14.0	ftp://ftp.ncbi.nlm.nih.gov/blast/executables/blast+/
BlobToolKit	4.3.7	https://github.com/blobtoolkit/blobtoolkit
BUSCO	5.4.3	https://gitlab.com/ezlab/busco
BUSCO	5.4.3 and 5.5.0	https://gitlab.com/ezlab/busco
bwa-mem2	2.2.1	https://github.com/bwa-mem2/bwa-mem2
Cooler	0.8.11	https://github.com/open2c/cooler
DIAMOND	2.1.8	https://github.com/bbuchfink/diamond
fasta_windows	0.2.4	https://github.com/tolkit/fasta_windows
FastK	427104ea91c78c3b8b8b49f1a7d6bbeaa869ba1c	https://github.com/thegenemyers/FASTK
GoaT CLI	0.2.5	https://github.com/genomehubs/goat-cli
Hifiasm	0.16.1-r375	https://github.com/chhylp123/hifiasm
HiGlass	1.11.6	https://github.com/higlass/higlass
HiGlass	44086069ee7d4d3f6f3f0012569789ec138f42b84aa44357826c0b6753eb28de	https://github.com/higlass/higlass
MerquryFK	d00d98157618f4e8d1a9190026b19b471055b22e	https://github.com/thegenemyers/MERQURY.FK
MitoHiFi	3	https://github.com/marcelauliano/MitoHiFi
MultiQC	1.14, 1.17, and 1.18	https://github.com/MultiQC/MultiQC
NCBI Datasets	15.12.0	https://github.com/ncbi/datasets
Nextflow	23.04.0-5857	https://github.com/nextflow-io/nextflow
PretextView	0.2	https://github.com/wtsi-hpag/PretextView
purge_dups	1.2.5	https://github.com/dfguan/purge_dups
samtools	1.16.1, 1.17, and 1.18	https://github.com/samtools/samtools
sanger-tol/genomenote	1.1.1	https://github.com/sanger-tol/genomenote
sanger-tol/readmapping	1.2.1	https://github.com/sanger-tol/readmapping
Seqtk	1.3	https://github.com/lh3/seqtk
Singularity	3.9.0	https://github.com/sylabs/singularity
TreeVal	1.0.0	https://github.com/sanger-tol/treeval
YaHS	1.2a.2	https://github.com/c-zhou/yahs

### Wellcome Sanger Institute – Legal and Governance

The materials that have contributed to this genome note have been supplied by a Darwin Tree of Life Partner. The submission of materials by a Darwin Tree of Life Partner is subject to the **‘Darwin Tree of Life Project Sampling Code of Practice’**, which can be found in full on the Darwin Tree of Life website here. By agreeing with and signing up to the Sampling Code of Practice, the Darwin Tree of Life Partner agrees they will meet the legal and ethical requirements and standards set out within this document in respect of all samples acquired for, and supplied to, the Darwin Tree of Life Project. 

Further, the Wellcome Sanger Institute employs a process whereby due diligence is carried out proportionate to the nature of the materials themselves, and the circumstances under which they have been/are to be collected and provided for use. The purpose of this is to address and mitigate any potential legal and/or ethical implications of receipt and use of the materials as part of the research project, and to ensure that in doing so we align with best practice wherever possible. The overarching areas of consideration are:

•      Ethical review of provenance and sourcing of the material

•      Legality of collection, transfer and use (national and international) 

Each transfer of samples is further undertaken according to a Research Collaboration Agreement or Material Transfer Agreement entered into by the Darwin Tree of Life Partner, Genome Research Limited (operating as the Wellcome Sanger Institute), and in some circumstances other Darwin Tree of Life collaborators.

## Data Availability

European Nucleotide Archive: *Eutrigla gurnardus* (grey gurnard). Accession number PRJEB64070; https://identifiers.org/ena.embl/PRJEB64070 ( Wellcome Sanger Institute, 2023). The genome sequence is released openly for reuse. The *Eutrigla gurnardus* genome sequencing initiative is part of the Darwin Tree of Life (DToL) project. All raw sequence data and the assembly have been deposited in INSDC databases. The genome will be annotated using available RNA-Seq data and presented through the Ensembl pipeline at the European Bioinformatics Institute. Raw data and assembly accession identifiers are reported in Table 1.

## Author information

Members of the Marine Biological Association Genome Acquisition Lab are listed here: https://doi.org/10.5281/zenodo.8382513.

Members of the Darwin Tree of Life Barcoding collective are listed here: https://doi.org/10.5281/zenodo.4893703.

Members of the Wellcome Sanger Institute Tree of Life Management, Samples and Laboratory team are listed here: https://doi.org/10.5281/zenodo.10066175.

Members of Wellcome Sanger Institute Scientific Operations: Sequencing Operations are listed here: https://doi.org/10.5281/zenodo.10043364.

Members of the Wellcome Sanger Institute Tree of Life Core Informatics team are listed here: https://doi.org/10.5281/zenodo.10066637.

Members of the Tree of Life Core Informatics collective are listed here: https://doi.org/10.5281/zenodo.5013541.

Members of the Darwin Tree of Life Consortium are listed here: https://doi.org/10.5281/zenodo.4783558.
