# Supplementary material for: The genome sequence of the grey gurnard, Eutrigla gurnardus (Linnaeus, 1758)
Source: Wellcome Open Res. 2024 Jun 11;9:307. [Version 1] doi: 10.12688/wellcomeopenres.22453.1 (PMC11377927; doi:10.12688/wellcomeopenres.22453.1)
Supplement: Peer review history

# Reviewer report: Reviewer response for version 1

**DOI:** 10.21956/wellcomeopenres.24740.r88060

**Published:** 2024-09-05

**Authors:** Fernando Cruz

**Affiliations:** 1 Centro Nacional de Análisis Genómico (CNAG), Barcelona, Spain

Congratultaions to the authors. After *Chelidonichthys spinosus*, this is the second chromosome-level reference genome available for the gunards (Family Triglidae) to date. The fEutGur1.1 assembly constitutes a valuable reference genome for this group and for Fish Genomics in general. The assembly meets by far the quality standards of the EBP and VGP.

**I have three comments and recommendations about this note:**

 1. Please specify that by gunards you refer to Family Triglidae in the text. Non experts in fish taxonomy will appreciate this.

 2. Please distinguish or split the total 24 missing joins or miss-joins into missing joins (to my knowledge "translocations) and mis-joins (to my knowledeg miss-assemblies).

 3. Please add the version of PretextView PretexMap etc to Table 3.

 Sincerely,

 Fernando Cruz

Are sufficient details of methods and materials provided to allow replication by others?

Yes

Is the rationale for creating the dataset(s) clearly described?

Yes

Are the datasets clearly presented in a useable and accessible format?

Yes

Are the protocols appropriate and is the work technically sound?

Yes

Reviewer Expertise:

Genome Assembly and Population Genomics

I confirm that I have read this submission and believe that I have an appropriate level of expertise to confirm that it is of an acceptable scientific standard.

# Reviewer report: Reviewer response for version 1

**DOI:** 10.21956/wellcomeopenres.24740.r89427

**Published:** 2024-08-13

**Authors:** Giacomo Bernardi

**Affiliations:** 1 University of California Santa Cruz, Santa Cruz, California, USA

This is a very straightforward report of a genome assembly.

 I have two very minor comments that pertain to the introduction:

- Fig 1. is the picture of the fish used for the genome not available?
- what does 'this' mean in the sentence : This genome represents the first of its kind for this Eutrigla gurnardus my interpretation is that there might be crytic species that are expected and 'this' is the genome of 'this' cryptic species?

Are sufficient details of methods and materials provided to allow replication by others?

Yes

Is the rationale for creating the dataset(s) clearly described?

Yes

Are the datasets clearly presented in a useable and accessible format?

Yes

Are the protocols appropriate and is the work technically sound?

Yes

Reviewer Expertise:

Fish genomics

I confirm that I have read this submission and believe that I have an appropriate level of expertise to confirm that it is of an acceptable scientific standard.

# Reviewer report: Reviewer response for version 1

**DOI:** 10.21956/wellcomeopenres.24740.r89419

**Published:** 2024-07-31

**Authors:** Elise Parey

**Affiliations:** 1 University College London, London, England, UK

This data note from the Darwin Tree of Life Consortium presents a high-quality chromosomes-scale genome assembly for the grey gurnard, as part of the consortium’s efforts to comprehensively sequence the UK eukaryotic biodiversity.

 The reported completeness and contiguity statistics support the excellent quality of this new genomic resource. I only have one main concern: the presented Hi-C contact map (Figure 5) does not show the correct scaffolding of contigs into chromosomes. On this contact map, no chromosomes are visible, only the diagonal. Inspecting the complete set of supplemental QC figures available online ( https://tolqc.cog.sanger.ac.uk/darwin/fish/Eutrigla_gurnardus/), the post-scaffolding Hi-C map "Juicebox YaHS Hi-C map: fEutGur1" appears more informative, with chromosomes clearly visible. However, a small mis-assembly can be suspected on this map: the first ~third of chr1 seems to have more contact with chr24 than with the rest of chr1. This might have been corrected during the described manual curation rounds, but it cannot be verified on the presented contact map.

 The methods are overall comprehensive. The Hi-C scaffolding steps could maybe be explained in more details: before scaffolding with YaHS, how were the Hi-C reads mapped and filtered? Finally, regarding the Quality Value (QV) it might be worth specifying that this relates to base accuracy (as opposed to structural accuracy), if I understood correctly.

Are sufficient details of methods and materials provided to allow replication by others?

Partly

Is the rationale for creating the dataset(s) clearly described?

Yes

Are the datasets clearly presented in a useable and accessible format?

Yes

Are the protocols appropriate and is the work technically sound?

Yes

Reviewer Expertise:

Genomics

I confirm that I have read this submission and believe that I have an appropriate level of expertise to confirm that it is of an acceptable scientific standard, however I have significant reservations, as outlined above.
